# Apoptosis and cell cycle aberrations in epithelial odontogenic lesions: An evidence by the expression of p53, Bcl-2 and Bax

**DOI:** 10.4317/medoral.22019

**Published:** 2018-02-25

**Authors:** Jefferson-da Rocha Tenório, Thalita Santana, Salomão-Israel-Monteiro-Lourenço Queiroz, Denise-Hélen-Imaculada-Pereira de Oliveira, Lélia-Maria-Guedes Queiroz

**Affiliations:** 1 DDs, Msc, PhD student, Faculty of Dentistry, University of São Paulo, São Paulo-SP, Brasil; 2 DDs, Msc, PhD student, Post-Graduation Program in Oral Pathology, Department of Dentistry, Federal University of Rio Grande do Norte, Natal – RN, Brasil; 3 DDs, Msc, PhD, Post-Graduation Program in Oral Pathology, Department of Dentistry, Federal University of Rio Grande do Norte, Natal – RN, Brasil; 4 DDs, Msc, PhD- Federal University of Rio Grande do Norte- Natal – RN, Brasil

## Abstract

**Background:**

Ameloblastoma (AMB), odontogenic keratocyst (OKC) and adenomatoid odontogenic tumor (AOT) are epithelial odontogenic lesions with diverse biologic profiles. Defects in regulation of apoptosis and cell cycle may be involved in the development and progression of those lesions, therefore we aimed to investigate the expression of Bcl-2, Bax and p53 to better understand the possible role of these proteins in AMBs, OKCs and AOTs.

**Material and Methods:**

The studied sample consisted of 20 AMBs, 20 OKCs and 20 AOTs. Immunohistochemistry technique was performed for the antibodies p53, Bcl-2 and Bax. Immunoreactivity was observed in the epithelial component and positive cells were counted in five fields (100x magnification). Statistical analysis was performed with Kruskal-Wallis and Spearman tests (*p*<0.05).

**Results:**

All lesions exhibited staining for the three studied proteins. There was no statistically significant associations between the expression of proteins and the lesions, however we identified a positive correlation between the expression of p53 and Bcl-2 (r = 0.200) and a negative correlation between p53 and Bax expressions (r = -0.100). In addition, p53 and Bax were similarly expressed between AMBs and OKCs. Bcl-2 was similarly expressed in AMBs and AOTs.

**Conclusions:**

Apoptosis regulatory proteins, as well as cell cycle proteins, are differently expressed in epithelial odontogenic lesions and their expression is possibly related to the biological behavior of AMB, OKC and AOT.

** Key words:**Odontogenic tumors, apoptosis, apoptosis regulatory proteins, p53 tumor suppressor protein, immunohistochemistry.

## Introduction

The development of the dental organ derives from epithelial cells of the primitive buccal cavity and ectomesenchymal cells of the neural crest, which induce the formation of ameloblasts and odontoblasts. The remnants from odontogenesis may give rise to the so-called odontogenic lesions, including cysts and tumors. Such lesions comprise approximately 2.5% of all lesions biopsied in dental offices (1).

The most prevalent odontogenic lesions are benign and derived from the odontogenic epithelium. Ameloblastoma (AMB) and Odontogenic Keratocyst (OKC) are the most common lesions and usually have a more aggressive clinical behavior. These lesions are important among maxillofacial lesions due to their clinical and histological heterogeneity (2). On the other hand, Adenomatoid Odontogenic Tumor (AOT) is known for its indolent clinical course and no tendency to relapse. This morphological and clinical behavior diversity is a reflection of the complex development of dental structures, since odontogenic lesions derive from aberrations in odontogenesis (3).

The growth rate of tissues is determined by proliferative activity and cell death. An imbalance between antiapoptotic proteins, such as Bcl-2 and Bax, can induce dysregulation of apoptosis (programmed cell death), which may lead to oncogenesis and tumour development. In addition, it has been proved that the inactivation of genes related to cell cycle regulation, such as p53, confers a selective advantage for tumor development, with subsequent impact on cellular activity alterations (4).

To better understand the role of apoptosis and cell proliferation in epithelial odontogenic lesions, we aimed to investigate the immunohistochemical expression of apoptosis proteins Bax and Bcl-2 and cell cycle protein p53 in AMB, OKC and AOT.

## Material and Methods

This study was approved by a local Research Ethics Committee (protocol number: 1.111.537).

-Samples

The samples consisted of 60 cases of epithelial odontogenic lesions, including 20 AMBs, 20 OKCs and 20 AOTs. The included AMBs were all multicystic, because it is the most common variant and represents the real biological behavior of this neoplasm. The OKCs associated with Nevoid Basal Cell Carcinoma Syndrome (NCBCS) were excluded from this study, because of its markedly different biologic behavior when compared to those that occur in isolated form. Lesions with an important inflammatory component were also excluded, since the presence of inflammation alters the expression of proteins associated with cellular proliferation, such as p53.

-Morphological analysis

Morphological analysis was performed on 5μm thick sections of paraffin embedded material, stained with hematoxylin and eosin (HE) and examined by light microscopy to reaffirm the histopathologic diagnosis as established by WHO (2017) (3).

-Immunohistochemistry

For immunohistochemical analysis, 3µm thick sections were mounted on organosilane-coated slides (3-aminopropyltriethoxsilane; Sigma Chemical Co., St. Louis,MO, USA). Antigen retrieval was performed in a Pascal pressure cooker with citrate buffer, pH 6.0, for three minutes. After treatment with normal serum, the sections were incubated with primary antibodies anti-Bcl-2 (124, Abcam, Cambrigde, MA, U.S.A.), anti-Bax (E63, Abcam, Cambrigde, MA, U.S.A.) and anti-p53 (DO7, Novocastra, Inc, Manhesset, NY, U.S.A) diluted respectively in 1:100, 1:500 and 1:1500 for 60 min. Following antigen retrieval, endogenous peroxidase was quenched with a 1:1 solution of methanol and 3% hydrogen peroxidase. Antibody was detected by immunoperoxidase staining using the dextran polymer-based signal enhancement technique (ADVANCE™, Dako, Carpinteria, CA, USA). For this reaction, diaminobenzidine chromogen was used. Negative control consisted of bovine serum albumin as replacement for the primary antibody. Lymphocytes present in the stromal tumor area, which have been previously shown to be strongly positive for the antibodies anti-Bcl-2, Bax and p53, served as internal positive control.

-Immunohistochemical assessment

All slides were examined independently by two observers. Immunostaining for all studied proteins was analyzed qualitatively under a light microscope (Olympus CH30, Olympus Japan Co., Tokyo, Japan) at a final magnification of 400x. Positive staining was defined as brown staining of epithelial cells and negative staining was the complete absence of staining. The staining distribution was analyzed semi-quantitatively using the following criteria adapted from Lee *et al.* (5): 0- absence of immunostaining; 1- (low) 1 to 10% positive cells; 2- (intermediate) 11-50% positive cells; and 3 (high)> 50% positive cells.

-Statistical analysis

For comparisons between protein expressions among the lesions, we used Kruskal-Wallis test and Spearman’s correlation test. The level of significance considered was 5% (*P* ≤ 0.05). Statistical analyses were performed using SPSS software package (IBM Corporation, Armonk, NY, USA).

## Results

-Adenomatoid odontogenic tumor 

**Figure 1:**
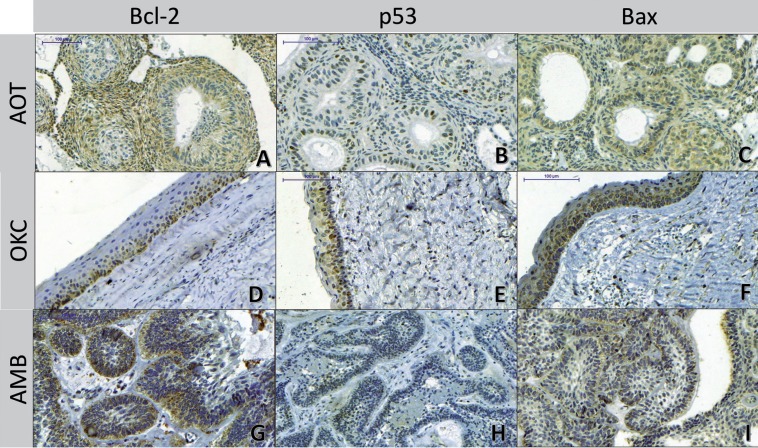
Immunohistochemical expression of Bcl-2, p53 and Bax proteins in AOTs, OKCs and AMBs (IHC, 200X). A) AOT - Immunostaining for Bcl-2 protein: positivity in tumor sheets; note that the duct-like cells exhibited no immunostaining for Bcl-2 protein; B) AOT - Immunostaining profile for p53 protein: low, diffuse and nuclear; C) AOT - Immunostaining profile for Bax protein: positivity along the duct-like structures. D) OKC - Immunostaining for Bcl-2 protein: low, cytoplasmic and diffuse; positive along the basal and parabasal cell layers; E) OKC - Immunostaining for p53 protein: note positivity in parabasal stratum and notable absence of marking in the basal cell layer; F) OKC - Immunostaining for the Bax protein: intense staining in all epithelial layers. G) AMB - Immunostaining for Bcl-2: positivity along the peripheral layer of the tumor follicles; Note that the central cells which resemble stellate reticulum of the enamel organ have no marking; H) AMB - Focal immunostaining for p53 protein; I) AMB - Immunostaining for Bax protein: positivity along the peripheral cells of the tumor follicles.

All studied AOTs showed positive expression for the analyzed proteins ( Table 1). This kind of tumor presented mostly moderate expression (score 2) for Bcl-2 protein (n = 11/55%), which was a predominantly cytoplasmic and diffuse expression among the tumor cells. The cells which form the duct-like structures were often negative for Bcl-2 protein (Fig. 1A). p53 showed low expression (score 1) (Fig. 1B) in most cases (n = 18/90%). Bax was intensely expressed (score 3) in half of the analyzed AOTs (n = 10/50%); cells that composed the duct-like structures were often immunostained for this protein (Fig. 1C).

**Table 1:**
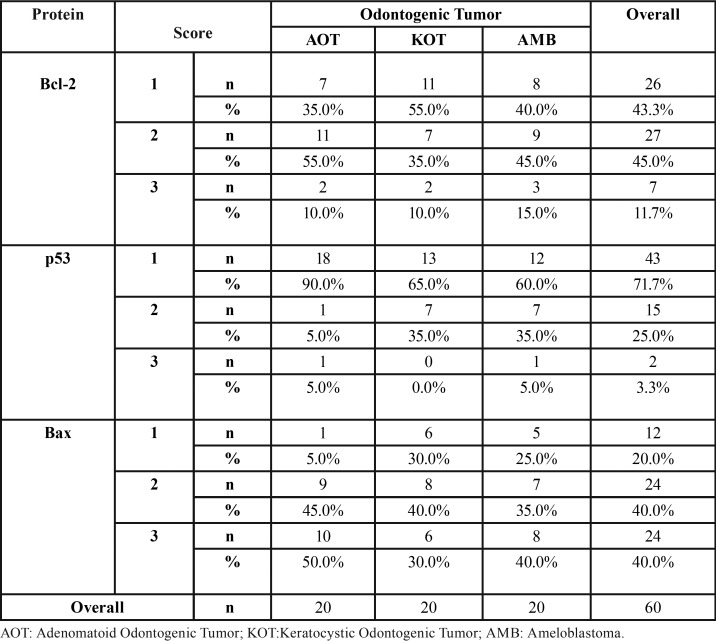
Frequency of immunostaining of Bcl-2, p53 and Bax according to the lesions.

-Odontogenic keratocyst

All OKCs showed positive expression for the studied proteins ( Table 1). OKCs presented mainly low expression (score 1) for the Bcl-2 protein (n = 11/55%). For this protein, we observed a cytoplasmic staining extending along the basal cell layer of the epithelial lining and sometimes to the parabasal cell layer. The cells of the upper layers were negative for Bcl-2 (Fig. 1D). Regarding p53, OKCs showed mainly low expression (score 1) of this protein (n = 11/55%). p53 was expressed in the cytoplasm of the basal cell layer and sometimes in the parabasal cell layer. The cells of the upper layers did not express p53 (Fig. 1E). OKCs exhibited variable immunostaining for the Bax protein, with a diffuse labeling through epithelial layers (Fig. 1F).

-Ameloblastoma

The AMBs were also positive for the studied proteins in all analyzed cases ( Table 1). The Bcl-2 protein showed predominantly moderate (score 2) (n = 9/45%) and low (score 1) expression (n = 8/40%); the immunolabeling was diffuse and occurred in cylindrical cells on the periphery of the follicles and epithelial cords (Fig. 1G). AMBs showed mainly low (score 1) (n = 16/60%) and diffuse immunostaining of p53 protein (Fig. 1H). Bax protein showed predominantly intense immunostaining (score 3) (n=8/40%), which occurred mainly on the periphery of epithelial nests (Fig. 1I).

-Statistical analysis

Comparing the immunoexpression of proteins between the lesions, we observed no statistically significant differences between scores ( Table 2). We observed positive correlations between Bcl-2 and p53 (r=0.200) and between Bcl-2 and Bax (r=0.197), and negative correlation between p53 and Bax (r=-0.100), however they were not statistically significant (*p* = 0.126; *p* = 0.131; *p* = 0.732, respectively).

**Table 2:**
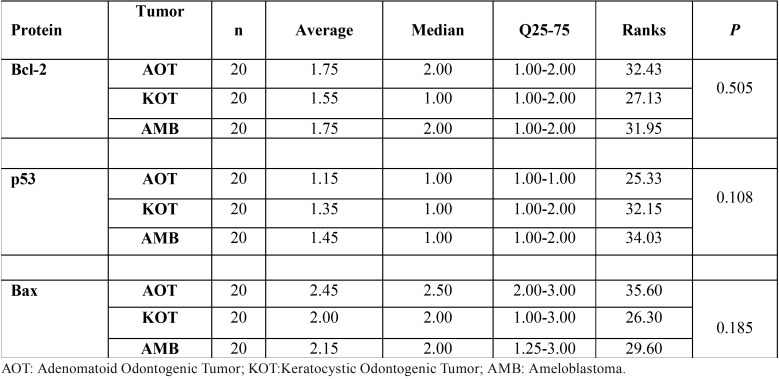
Evaluation of the differences between the lesions according to Bcl-2, p53 and Bax immunostaining.

## Discussion

Odontogenic lesions are a heterogeneous group of disorders that can be classified as hamartomas, cysts or benign and malignant neoplasms, with a quite variable biological behavior and aggressiveness. Those lesions are considered rare and occur only in the maxillary bones, arising due to aberrations during the differentiation of dental structures. In 2017, the World Health Organization (WHO) reclassified the keratocystic odontogenic tumor back into the odontogenic cysts category (OKC)(3), however this is still a controversial matter, since 85% of OKCs related to the Nevoid basal cell carcinoma syndrome (NBCCS) and 30% of non-syndromic cases present mutations in the PTCH gene (6). Also, this lesion is well known for its aggressiveness and recurrence rate after treatment (2).

The development of odontogenic lesions is related to alterations in oncogenes, tumor suppressor genes, oncoviruses, cell cycle controllers, DNA repair genes, apoptosis related elements, among others (7). Tumor suppressor genes play a key role in tumor development and p53 is one of the most important altered genes in oncogenesis since, under normal conditions, its products are responsible for genomic equilibrium through induction of apoptosis and cell cycle blocking (8). Likewise, oncogenesis involves loss of balance between regulators of cell proliferation and apoptosis. Cell death by apoptosis plays a key physiological role in tissue development and homeostasis, and dysregulation in apoptosis is also responsible for events such as carcinogenesis, tumor progression and resistance of tumor cells to chemotherapy (9).

In the present study, we observed p53 protein expression in all cases of AMBs, OKCs and AOTs. This finding is in agreement with Garg *et al.* (7) who confirmed the presence of this protein in several odontogenic lesions, including AMBs, ameloblastic carcinomas and ameloblastic fibrosarcoma. However, it contrasts with the inferences of Sauk *et al.* (10), who believe that the expression of p53 is only an occasional finding in odontogenic lesions. Besides, p53 was highly expressed in OKCs and AMBs, while AOTs presented predominantly mild immunolabeling. Although this was not statistically significant, this observation corroborates with the findings of Salehinejad *et al.*(11), which showed a higher positivity index for p53 in AMBs than in AOTs. For this reason, we can infer that there is a greater dysregulation in cell cycle control in lesions with a more aggressive biological behavior, such as AMB and OKC. Still, p53 protein immunoexpression was similar between AMBs and OKCs, which was also previously observed (12). This could reinforce the neoplastic phenotype theory in OKCs.

Another differential characteristic of p53 expression was its marked presence in the parabasal cell layer of OKCs, with evident absence of immunopositivity in the basal cell layer. This characteristic was also observed by Sajeevan *et al.* (13) in an evaluation of p53 and PCNA expression in OKCs and radicular cysts. On the other hand, Deyhimi and Hashemzade (14) found a higher expression of these proteins in the basal cell layer of OKCs and orthokeratinized cysts. The explanation for this fact may be related to the lowest stage of differentiation of basal cell layer in these lesions, as well as a longer G1 phase in the parabasal cell layer, which allows a greater protein detection (13).

Regarding Bcl-2 and Bax expressions, all cases evaluated showed some degree of immunoreactivity to these proteins. This finding was also observed by different studies that investigated the imbalance in cellular apoptosis in different tumors (15-17). We observed that Bcl-2 was similarly expressed in AOTs and AMBs, with a score of 2 (moderate) in most of the analyzed cases. On the other hand, Razavi *et al.* (15) reported an increased expression of Bcl-2 and Ki-67 in AMBs when compared to AOTs, which could imply less cell cycle stability and greater aggressiveness to AMBs, when compared to AOTs. Furthermore, we observed reduced expression of Bcl-2 in OKCs, compared to AMBs and AOTs, which differs from the findings of Amaral *et al.* (18) that demonstrated, through the TUNEL method, similar apoptotic index in AMBs and OKCs. The wide variety of techniques for evaluating apoptosis as well as the diverse immunohistochemical protocols may explain the differences in these findings.

Bcl-2 was expressed predominantly in the peripheral cells of the follicles and plexiform arrangements of the AMBs, corroborating with other findings (19). The expression of Bcl-2 in the outer layer of AMB cells may suggest not only its proliferative activity, but also the inhibition of cell death, just as it occurs in the dental germ, characteristics that reflect on the growth potential of this neoplasm (19). Regarding OKCs, Bcl-2 was observed mainly in the basal cell layer of the epithelial lining, which also has been observed before (16). The lack of expression in the upper layers may be due to the decrease in the cell ability to divide and to the end of its life cycle (16). In addition, this characteristic immunoexpression may point to the abnormal control of the cell cycle in these lesions.

Comparing Bax expression between lesions in AMBs and OKCs, we observed quite similar immunostaining. This was also reported by Soluk Tekkeşin *et al.* (4), that evaluated the expression of this protein in AMBs, OKCs and radicular cysts. Bax was also reported to have increased expression in solid AMBs, when compared to the unicystic variant (19). According to the authors, this finding could explain the spectrum of biological behavior of this lesion. On the other hand, Rangiani and Motahhary (20) did not find a statistically significant differences in Bax expression between OKCs and orthokeratinized odontogenic cysts; The authors suggest that this protein studied alone does not reflect on the biological behavior of OKCs.

Moreover, in the present study we identified a positive correlation between the expression of p53 and Bcl-2, as well as a negative correlation between p53 and Bax, although this was not statistically significant. Nevertheless, we can infer that there is an important relation between odontogenic lesions that overexpress p53 with the increase in antiapoptotic factors expression (Bcl-2), as well as a reduction in the expression of pro-apoptotic factors (Bax). This could be explained by the fact that in normal conditions, p53 contributes to the induction of apoptosis, acting directly at the mitochondrial level. However, in adverse conditions, such as in neoplasms, there is a greater expression of antiapoptotic factors, which favors cell survival and escape mechanisms of cell cycle control (8).

In conclusion, we observed that apoptosis regulatory proteins Bax and Bcl-2 and cell cycle protein p53 are differently expressed in the epithelial odontogenic lesions AMB, OKC and AOT, which is possibly related to their diverse biological behavior. Further investigation is needed to elucidate the roles of these proteins in odontogenesis and development of cystic and neoplastic odontogenic lesions.
